# Cross-sectional analysis of primary care clinics’ policies, practices, and availability of patient support services during the COVID-19 pandemic

**DOI:** 10.1186/s12913-024-10660-6

**Published:** 2024-03-05

**Authors:** Kendra L. Ratnapradipa, Runqiu Wang, Josiane Kabayundo, Walter Marquez Lavenant, Eleanore Nelson, Muskan Ahuja, Ying Zhang, Hongmei Wang

**Affiliations:** 1 Department of Epidemiology, University of Nebraska Medical Center, 984395 Nebraska Medical Center, Omaha, NE 68198-4395 USA; 2 Department of Biostatistics, University of Nebraska Medical Center, Omaha, NE USA; 3 Department of Health Services Research & Administration, University of Nebraska Medical Center, Omaha, NE USA

**Keywords:** Health care/services, Accessibility, Rural-urban disparities, “no show” or missed appointments, COVID-19

## Abstract

**Background:**

Healthcare accessibility and utilization are important social determinants of health. Lack of access to healthcare, including missed or no-show appointments, can have negative health effects and be costly to patients and providers. Various office-based approaches and community partnerships can address patient access barriers.

**Objectives:**

(1) To understand provider perceptions of patient barriers; (2) to describe the policies and practices used to address late or missed appointments, and (3) to evaluate access to patient support services, both in-clinic and with community partners.

**Methods:**

Mailed cross-sectional survey with online response option, sent to all Nebraska primary care clinics (*n* = 577) conducted April 2020 and January through April 2021. Chi-square tests compared rural-urban differences; logistic regression of clinical factors associated with policies and support services computed odds ratios (OR) and 95% confidence intervals (CI).

**Results:**

Response rate was 20.3% (*n* = 117), with 49 returns in 2020. Perceived patient barriers included finances, higher among rural versus urban clinics (81.6% vs. 56.1%, *p* =.009), and time (overall 52.3%). Welcoming environment (95.5%), telephone appointment reminders (74.8%) and streamlined admissions (69.4%) were the top three clinic practices to reduce missed appointments. Telehealth was the most commonly available patient support service in rural (79.6%) and urban (81.8%, *p* =.90) clinics. Number of providers was positively associated with having a patient navigator/care coordinator (OR = 1.20, CI = 1.02–1.40). For each percent increase in the number of privately insured patients, the odds of providing legal aid decreased by 4% (OR = 0.96, CI = 0.92-1.00). Urban clinics were less likely than rural clinics to provide social work services (OR = 0.16, CI = 0.04–0.67) or assist with applications for government aid (OR = 0.22, CI = 0.06–0.90).

**Conclusions:**

Practices to reduce missed appointments included a variety of reminders. Although finances and inability to take time off work were the most frequently reported perceived barriers for patients’ access to timely healthcare, most clinics did not directly address them. Rural clinics appeared to have more community partnerships to address underlying social determinants of health, such as transportation and assistance applying for government aid. Taking such a wholistic partnership approach is an area for future study to improve patient access.

**Supplementary Information:**

The online version contains supplementary material available at 10.1186/s12913-024-10660-6.

## Introduction

Lack of regular access to health care can have negative health effects, particularly for patients with chronic conditions [1]. Insurance status and cost of care are two widely recognized barriers to accessing care [2]. Limiting needed care is one mechanism for addressing high medical costs [3] and can include missed appointments. Missed appointments are generally classified as no-shows or broken appointments when a patient fails to attend as scheduled or cancels with less than 24-hour notice. If more than 24 h, the appointment is considered canceled [4, 5].

Missed appointments may result in loss of revenue, inefficiency, and poor productivity for healthcare providers [4, 6, 7]. A higher rate of no-shows has been observed in rural clinics and clinics that serve minority populations [4, 6–8]. An estimated 3.6 million Americans missed at least one medical appointment during the year due to transportation barriers [1]. Other factors associated with no-shows include the patient forgetting the appointment, longer lead time (i.e., time between identified need and receipt of care), prior history of no-shows, relationship with healthcare providers, education, and income [4, 9].

Providers utilize different approaches to reduce no-shows, with most emphasizing patient behaviors and healthcare performance rather than underlying social determinants of health that the patient may be experiencing. Methods employed include reminder messages, overbooking, and patient penalization [10]. Overbooking is defined as booking multiple patients in a common time slot, and it works efficiently when the no-show rate is high [11]. Practicing overbooking may reduce the rate of no-shows, improve healthcare utilization, and increase physicians’ productivity [11, 12]. This may be due, at least in part, to allowing clinics to schedule patients sooner, because longer wait periods between initial contact and appointment date have been consistently associated with no-shows [13, 14]. However, when healthcare providers overbook schedules to reduce down-time from missed appointments, this practice impacts other patients in the system, including those who keep their appointments [15, 16]. Thus, missed appointments may affect the scheduling system, resulting in longer wait times and patient dissatisfaction [5, 17, 18].

Existing literature focuses on barriers that predict no-show rates for specific conditions, subpopulations, practice location, or broad conceptualizations of healthcare access. However, there is still a critical need to understand what support services are available to patients to address social determinants of health, the eligibility requirements, and the impact on short- and long-term health outcomes. Nebraska is a primarily rural state, with only 26.7% of its need for primary care currently met [19]. The aims of our study were to address the first of these issues, mainly, (1) to understand primary care providers’ perceptions of the barriers to care faced by their patients, as this may motivate providers to partner with community organizations to address such perceived needs; (2) to describe the policies and practices used to address late or missed appointments, and (3) to evaluate availability of patient support services, both in-clinic and with community partners.

## Methods

The study protocol was screened by the University of Nebraska Medical Center Institutional Review Board prior to data collection. Because the unit of analysis was health care practice/facility, the study was determined to be quality improvement and did not constitute human subject research as defined at 45CFR46.102. All methods were carried out in accordance with relevant guidelines and regulations. A cover letter described the study and completion of the voluntary survey represented informed consent.

### Study design and participants

This was a cross-sectional survey sent to all 577 primary care clinics in Nebraska excluding satellite practice locations. Primary care specialties were defined as follows: family medicine, general practice, internal medicine, pediatrics, obstetrics/gynecology, women’s health, adolescent medicine, adult medicine, and gerontology/geriatric medicine. The University of Nebraska Medical Center’s Health Professions Tracking Service (HPTS) maintains a statewide database of licensed healthcare provider information organized by clinic/facility.

### Survey instrument and variables

The 4-page survey was developed for this study based on existing literature about barriers and access to care [18, 20] with additional questions designed by the study team to capture data associated with clinic charactersitcs in the preceeding year. The survey addressed the following topics: patient access, patient resources, policies and procedures, clinic characteristics in 2019, and information about which office staff or clinician completed the questionnaire (see Additional file 1). Following an unanticipated project delay due to COVID-19, the survey was updated between rounds 1 and 2 to simplify questions (reporting of aggregated patient counts/percents) and to gather information about clinic characteristics for 2019 and 2020, as well as including an additional section about the impacts of COVID-19, which is not included in this analysis (see Additional file 2). Revisions also included building an online version of the survey in Research Electronic Data Capture (REDCap).

*Clinic characteristics* were based on the HPTS database, which includes the number of practitioners at the primary location and satellite locations by the practitioner’s primary specialty, and the facility’s location and contact information. We used the total number of practitioners as a measure of clinic size and created a variable to indicate clinics with affiliated satellite location(s) (yes/no). This information was merged with returned survey responses using unique clinic identification numbers. ZIP code of clinic location was merged with Rural-Urban Commuting Area (RUCA) codes dichotomized as urban (codes 1–3) and rural (codes 4–10). Survey-based clinic information included use of electronic medical records (yes/no) and if so, whether they were used to flag vaccine and cancer screening status. We also asked about the approximate number of patients seen at the practice and the percent of patients by insurance status (Medicaid, Medicare, private insurance, uninsured or self-pay).

*Perceived patient barriers* was a list of common patient barriers (i.e., clinic hours, finances, language, time off work, appointment not soon enough, forgot to schedule, dependent care, transportation, wait times, other), asking “What factors seem to most imparct your patients’ ability to receive timely access to health care?” For round 1, these were rank-ordered but recoded as rank 1–5 = yes and rank 6–9 = no to correspond to question rewording in round 2.

*Patient resources* asked respondents to indicate if the following services were available on-site, through a community partner, or not available: patient navigator/care coordinator; telehealth; social work; legal aid; assistance applying for government aid (e.g., Medicaid, Medicare, Supplemental Nutritional Assistance Program); transportation; dependent care.

*Policies and procedures* included a yes/no checklist of procedures and practices that may be used to reduce missed appointments: overbooking; set-aside walk-in appointments; increasing provider capacity in past 2 years; motivational interviewing; contingency management; telephone, text, email, and mailed scheduling reminders and appointment reminders; streamlined admission; welcoming environment; extended evening and weekend hours; fees for late arrival, late cancellation and no-show; dropped from service for missed appointments (if yes, specify number). Open response follow-up identified policies for late arrival and missed appointments. We also asked how much missed appointments were a problem for the practice (not at all, slightly, somewhat, very much).

### Data collection

HPTS mailed a cover letter, the survey, and a postage paid return business envelope on 17 April 2020. Due to the worsening COVID-19 crisis in the state and some early returns declining participation citing the healthcare crisis, data collection was put on hold. During the interval, an online version of the revised survey was built in REDCap to provide additional response flexibility for participants and to decrease potential for data misclassification.

Data collection recommenced mid-January 2021. HTPS sent a revised cover letter that contained a web address for those opting to complete the survey online, the revised survey, and a business reply envelope. These were sent only to providers who did not respond during the initial round of data collection in an attempt to reduce burden on providers during the ongoing pandemic. During April-May 2021, researchers called all clinics that had not responded and asked if they would complete the survey over the phone or online.

### Data cleaning and management

Data were initially entered in Excel (Round 1, *n* = 49), but were migrated to REDCap beginning with Round 2, at which time Round 1 data were re-entered. Paper-based returns were double entered as a quality control measure. Any discrepancies or data irregularities were discussed as a team and adjudicated by a third researcher. Following resolution, duplicate entries were removed. An indicator variable for the round of data collection was added and we combined individual rounds of data into a workable analysis file. Survey data were merged with HTPS clinical data and RUCA codes. Several questions, particularly related to the clinical population, had significant missingness and were excluded from analysis. The 17 online responses (14.5%) were missing clinic identifiers and unable to be classified as rural/urban. Otherwise, missingness was addressed by case-wise exclusion.

### Analysis

Descriptive statistics included count and percent with Chi-square comparisons for urban-rural differences for patient barriers, resources, policies and procedures, and clinic characteristics. Univariate analysis examined differences between urban and rural areas in perceived barriers to care, burden of missed appointments, and supportive services. Each type of support service was modeled separately, and all were modeled for any availability (onsite or partner) as well as on-site only. Independent variables were jointly modeled and included estimated percent of patients using Medicaid, Medicare, and private insurance (patients could use more than one type of insurance), number of practitioners at primary location, satellite location, and rural-urban status. Data are presented as odds ratios (OR) with 95% confidence intervals (CI) and *p*-values, with *p*-values < 0.05 considered statistically significant using 2-tailed testing. Statistical analyses were conducted using SAS (version 9.4) and RStudio (version 4.0.4).

## Results

**Fig. 1:**
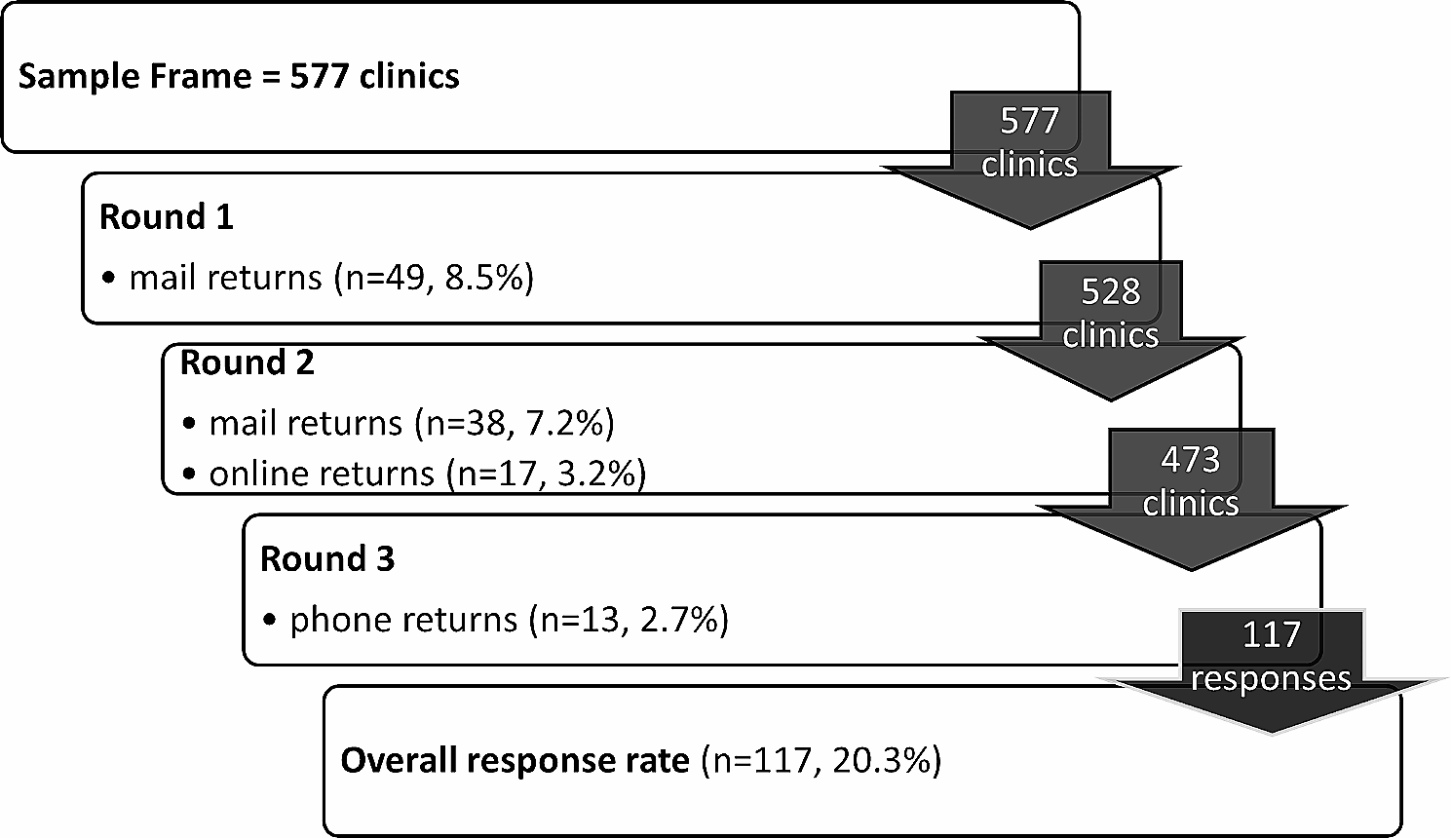
Survey response rates by round of data collection

The overall response rate was 20.3% (*n* = 117), with 49 mailed returns in 2020 and 38 mailed, 17 online, and 13 phone responses in 2021 (Fig. 1). Most responses (54%) were from rural areas. A sensitivity analysis examined non-response bias and is available in an additional file (See Additional file 3). Rural-urban status was the only clinical characteristic that was significantly different between clinics that participated in the survey and those that did not respond (*p* <.001), with rural clinics being more likely to respond than urban clinics. Overall, 64.7% of responding clinics were classified as family medicine; 85% of rural clinics were family medicine (Table 1). More than half (56%) of patients seen at urban clinics were privately insured, while Medicare was the most frequent insurance type for rural clinics (40%).

**Table 1:** Summary of clinic characteristics

Characteristic	Missing	Overall *n* = 117	Rural *n* = 55	Urban *n* = 44
Has satellite location, n (%)	18	29 (24.8)	16 (29.1)	13 (29.5)
Family medicine specialty, n (%)	18	67 (57.3)	47 (85.5)	20 (45.5)
Number of providers at primary location, mean (SD)	18	4.2 (4.1)	4.0 (3.1)	4.4 (5.1)
Percent Medicaid, mean (SD)	50	23.5 (21.6)	21.1 (19.1)	22.9 (21.8)
Percent Medicare, mean (SD)	59	36.1 (25.7)	40.4 (24.2)	29.4 (29.1)
Percent private insurance, mean (SD)	45	45.1 (27.1)	38.3 (22.1)	56.3 (29.9)

### Perceived patient barriers

**Fig. 2:**
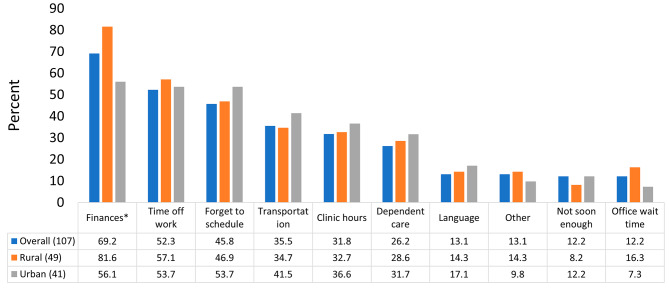
Percent of Nebraska primary care clinics indicating perceived barriers to patient receipt of timely care Notes: *p <.05

The most frequently perceived factors impacting patients’ timely access to care (overall, rural, and urban clinics) were financial concerns (69.2%, 81.6%, and 56.1%; *p* =.009), the inability of patients to get time off work (52.3%, 57.1%, and 53.7%; *p* =.72), and forgetting to schedule (45.8%, 46.9%, and 53.7%; *p* =.53) (Fig. 2). In a sensitivity analysis of round 1 ranked responses (results not shown), finances, inability to take time off from work, and dependent care were the most frequently perceived barriers.

### Clinic procedures and practices to reduce missed appointments

**Fig. 3:**
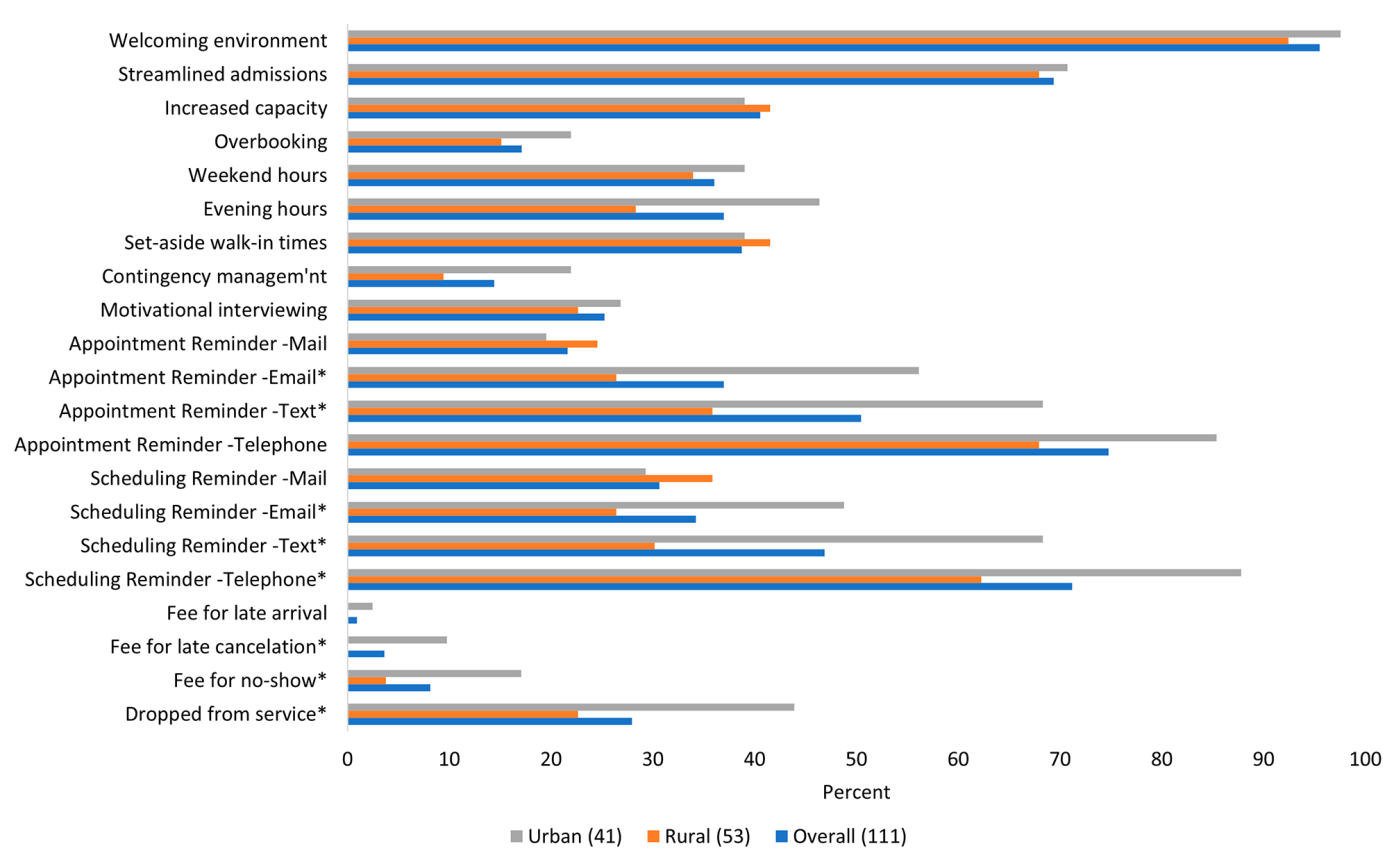
Policies and practices employed by Nebraska primary care clinics to reduce missed appointments Notes: *p <.05

Most clinics did not consider missed appointments to be much of a problem, with 20.5% indicating it was not at all a problem, and 53.9% indicating a slight problem. We ran additional analysis to determine if clinical factors (Table 1) or patient support services (Table 2) were associated with missed behaviors being problematic; none were statistically significant (results not shown). Over half (54.7%) the responding clinics had a policy to address late arrivals, and 44.4% had a policy for missed appointments. Although uncommon, urban clinics were statistically more likely than rural clinics to use punitive measures such as dropping patients from service (43.9% vs. 22.6%, *p* =.03) and charging fees for missed appointments (17.1% vs. 3.8%, *p* =.03) or late cancelations (9.8% vs. 0%, *p* =.02). There was wide variability in the procedures and practices employed by clinics to minimize missed appointments (Fig. 3) Nearly all clinics (95.5%) reported having a welcoming environment, 74.8% use telephone appointment reminders, and 69.4% have streamlined admissions. Less than half the clinics increased provider capacity in the past two years (40.5%), set aside walk-in times (38.7%), had evening (36.9%) or weekend hours (36.0%), used motivational interviewing (25.2%), overbooked (17.1%), or used contingency management (14.4%). Urban versus rural clinics were statistically more likely to use telephone scheduling reminders (87.8% vs. 62.3%, *p* =.005), text appointment (68.3% vs. 35.9%, *p* =.002) and scheduling (68.3% vs. 30.2%, *p* <.001) reminders, and email appointment (56.1% vs. 26.4%, *p* =.004) and scheduling (48.8% vs. 26.4%, *p* =.03) reminders.

**Table 2:** Availability of patient support services, by rural-urban status

Patient Services	Location	Overall *N* = 116	Rural *N* = 54	Urban *N* = 44	***P***-value
Telehealth options	Onsite	92 (79.3)	42 (77.8)	35 (79.5)	0.97
	Partner	2 (1.7)	1 (1.9)	1 (2.3)	
	Not Available	18 (15.5)	9 (16.7)	6 (13.6)	
Patient navigator or care coordinator	Onsite	51 (44.0)	25 (46.3)	18 (40.9)	0.79
	Partner	8 (6.9)	5 (9.3)	3 (6.8)	
	Not Available	54 (46.6)	23 (42.6)	21 (47.7)	
Application assistance	Onsite	28 (24.1)	14 (25.9)	8 (18.2)	0.06
	Partner	30 (25.9)	17 (31.5)	6 (13.6)	
	Not Available	53 (45.7)	22 (40.7)	27 (61.4)	
Social Work	Onsite	19 (16.4)	11 (20.4)	4 (9.1)	0.01**
	Partner	36 (31.0)	21 (38.9)	9 (20.5)	
	Not Available	57 (49.1)	22 (40.7)	28 (63.6)	
Transportation services	Onsite	11 (9.5)	6 (11.1)	3 (6.8)	0.10
	Partner	38 (32.8)	22 (40.7)	9 (20.5)	
	Not Available	63 (54.3)	25 (46.3)	30 (68.2)	
Legal Aid	Onsite	1 (0.9)	1 (1.9)		0.78
	Partner	28 (24.1)	13 (24.1)	9 (20.5)	
	Not Available	80 (69.0)	37 (68.5)	32 (72.7)	
Dependent care services	Onsite	0	0	0	0.31
	Partner	14 (12.1)	9 (16.7)	3 (6.8)	
	Not Available	96 (82.8)	42 (77.8)	39 (88.6)	

*p <.05, **p <.01

### Patient support services

Patient support services were considered as available on-site or with a partner. The only patient support service available on-site at more than half the clinics was telehealth (79.3%) (Table 2). Patient navigation or care coordination was available at 44.0% of clinics, and assistance applying for government aid programs was available on-site at 24.1% of clinics. Patient support services available through partners included transportation services (32.8%), social work (31.0%), assistance applying for aid programs (25.9%), legal aid (24.1%), and dependent care (12.1%). Support services (onsite or with partner) were more common in rural compared to urban clinics for all types of services surveyed. A significantly higher proportion of rural than urban clinics offered social work (59.3% vs. 29.6%; *p* =.004), assistance applying for government aid (57.4% vs. 31.8%; *p* =.03), and transportation services (51.9% vs. 27.2%; *p* =.04).

Logistic regression results for each type of support service are reported based on any availability (onsite and/or partner) first, followed by analysis of on-site only (see Table 3). For each additional provider at the main clinic location, the odds of having a patient navigator or care coordinator increased by 20% (OR 1.20; 95% CI 1.02–1.40). Odds of providing social work services were 84% less for urban vs. rural clinics (OR 0.16; 95% CI 0.04–0.67). For each percent increase in privately insured patients, the odds of providing legal aid decreased by 4% (OR 0.96; 95% CI 0.92-1.00). Odds of providing assistance applying for government aid were 78% less for urban vs. rural clinics (OR 0.22; 95% CI 0.06–0.90).

**Table 3:** Multivariate logistic regression of availability of patient support services

Support Service	Variable	OR (95% CI)	***P***-value
**Available On-Site or with Partner**			
Patient navigator or care coordinator	Urban (vs. Rural)	0.80 (0.24, 2.75)	0.73
	Number of providers	1.20 (1.02, 1.40)	0.03*
	Satellite	1.61 (0.44, 5.91)	0.48
	% Medicaid	1.00 (0.97, 1.03)	1.00
	% Medicare	0.99 (0.96, 1.02)	0.48
	% Private insurance	0.99 (0.95, 1.02)	0.41
Telehealth options	Urban (vs. Rural)	0.33 (0.07, 1.50)	0.15
	Number of providers	1.13 (0.92, 1.39)	0.24
	Satellite	6.24 (0.74, 52.67)	0.09
	% Medicaid	0.96 (0.91, 1.01)	0.11
	% Medicare	0.97 (0.92, 1.01)	0.12
	% Private insurance	1.01 (0.96, 1.06)	0.74
Social Work	Urban (vs. Rural)	0.16 (0.04, 0.67)	0.01**
	Number of providers	1.09 (0.96, 1.24)	0.19
	Satellite	1.00 (0.25, 4.04)	1.00
	% Medicaid	1.02 (0.98, 1.06)	0.39
	% Medicare	0.98 (0.95, 1.01)	0.19
	% Private insurance	0.97 (0.93, 1.01)	0.11
Legal Aid	Urban (vs. Rural)	1.34 (0.29, 6.20)	0.71
	Number of providers	1.06 (0.92, 1.23)	0.42
	Satellite	0.77 (0.15, 3.96)	0.75
	% Medicaid	1.01 (0.97, 1.05)	0.61
	% Medicare	0.98 (0.95, 1.01)	0.23
	% Private insurance	0.96 (0.92, 1.00)	0.05*
Application Assistance	Urban (vs. Rural)	0.22 (0.06, 0.90)	0.04*
	Number of providers	1.05 (0.93, 1.20)	0.44
	Satellite	3.40 (0.82, 14.17)	0.09
	% Medicaid	1.03 (0.98, 1.08)	0.23
	% Medicare	1.00 (0.97, 1.03)	0.91
	% Private insurance	0.98 (0.95, 1.02)	0.32
Transportation Services	Urban (vs. Rural)	0.25 (0.06, 1.04)	0.06
	Number of providers	1.04 (0.91, 1.18)	0.58
	Satellite	3.21 (0.84, 12.36)	0.09
	% Medicaid	1.03 (0.98, 1.07)	0.23
	% Medicare	0.99 (0.96, 1.02)	0.50
	% Private insurance	0.99 (0.96, 1.03)	0.65
Dependent care	Urban (vs. Rural)	0.27 (0.02, 3.40)	0.31
	Number of providers	1.00 (0.79, 1.27)	0.99
	Satellite	4.50 (0.61, 33.12)	0.14
	% Medicaid	0.98 (0.92, 1.05)	0.55
	% Medicare	1.00 (0.95, 1.05)	0.98
	% Private insurance	1.00 (0.95, 1.07)	0.90
**Available On-Site Only**			
Patient navigator or care coordinator	Urban (vs. Rural)	1.13 (0.32, 4.01)	0.86
	Number of providers	1.19 (1.02, 1.38)	0.02*
	Satellite	0.54 (0.14, 2.08)	0.37
	% Medicaid	1.01 (0.97, 1.04)	0.78
	% Medicare	1.00 (0.97, 1.03)	0.84
	% Private insurance	0.97 (0.94, 1.00)	0.08
Telehealth options	Urban (vs. Rural)	0.28 (0.06, 1.23)	0.09
	Number of providers	1.13 (0.93, 1.36)	0.22
	Satellite	6.55 (0.76, 56.04)	0.09
	% Medicaid	0.95 (0.90, 1.01)	0.09
	% Medicare	0.97 (0.93, 1.01)	0.13
	% Private insurance	1.00 (0.96, 1.06)	0.87
Social Work	Urban (vs. Rural)	0.20 (0.02, 1.74)	0.14
	Number of providers	1.19 (1.00, 1.41)	0.05
	Satellite	1.64 (0.30, 9.08)	0.57
	% Medicaid	1.02 (0.97, 1.07)	0.38
	% Medicare	1.02 (0.98, 1.07)	0.33
	% Private insurance	1.00 (0.94, 1.06)	0.93
Legal Aid	Urban (vs. Rural)	< 0.001 (< 0.001, > 999.99)	0.39
	Number of providers	430.64 (< 0.001, > 999.99)	0.86
	Satellite	< 0.001 (< 0.001, > 999.99)	0.74
	% Medicaid	3.38 (0.38, 30.51)	0.28
	% Medicare	0.81 (0.00, 379.46)	0.95
	% Private insurance	0.09 (< 0.001, 61.67)	0.47
Application Assistance	Urban (vs. Rural)	0.49 (0.09, 2.60)	0.40
	Number of providers	1.01 (0.85, 1.19)	0.93
	Satellite	4.78 (1.08, 21.11)	0.04*
	% Medicaid	1.02 (0.98, 1.07)	0.30
	% Medicare	1.00 (0.96, 1.03)	0.75
	% Private insurance	0.98 (0.94, 1.02)	0.34
Transportation Services	Urban (vs. Rural)	0.22 (0.01, 3.52)	0.29
	Number of providers	1.06 (0.83, 1.36)	0.64
	Satellite	3.19 (0.37, 27.12)	0.29
	% Medicaid	1.02 (0.98, 1.07)	0.30
	% Medicare	1.00 (0.96, 1.04)	0.88
	% Private insurance	0.99 (0.93, 1.06)	0.75

*p <.05, **p <.01

When considering only services provided on-site, for each additional provider at the main clinic location the odds of having a patient navigator or care coordinator increased by 19% (OR 1.19; 95% CI 1.02–1.38). Odds of assistance applying for government aid were 4.78 times higher for clinics with a satellite office (OR 4.78; 95% CI 1.08–21.11). A sensitivity analysis (not shown) compared the number of support services offered on-site (0 vs. 1 or more). Only the number of practitioners at the primary site was significant (OR 1.17; 95% CI 1.01–1.35).

## Discussion

Study results revealed that clinic policies, practices, and support services do not align well with addressing perceived patient barriers to accessing timely healthcare services. Finance was the most frequently identified perceived patient barrier, but only half the clinics offered assistance to patients to apply for government aid (24% on-site, 26% partnering with outside organization). Unsurprisingly, larger clinics were better able to provide additional support services compared to smaller clinics. Costs for patient navigation/coordination and social work are not directly billable as fee-for-service patient encounters, and therefore are better supported by clinics that use a patient-centered medical home or integrated care model [21], such as those affiliated with a hospital or large healthcare network. Additionally, patient ability to take time off work was the second most frequently indicated barrier, but only 38.7% of clinics had walk-in schedules, 35% had extended evening hours, and 34% had weekend hours.

One aspect of healthcare access is missed appointments. Previous studies have found medical cost was associated with patient no-shows [5]. Insured patients have a lower no-show rate compared to those who pay out of pocket; moreover, patients with low socio-economic status are more likely to miss healthcare appointments [8]. Other factors associated with missing a healthcare appointment include dependent care and not being able to get time off work [22]. Parents may miss healthcare appointments because they cannot afford daycare. Moreover, patients who work for companies that do not allow them to take paid off time to get medical services are more likely to miss their healthcare appointments [22]. None of the clinics that participated in our study had dependent care services available on-site, and only 12% partnered with outside organizations to provide such services.

Transportation is also associated with missed appointments [23] and access to care [14]. Patients who do not own a car or must rely on public transportation are more likely to miss their healthcare appointments, with as many as 25% of participants missing appointments due to transportation barriers [23]. Bus users were twice as likely to miss their appointments compared to car users [24]. Because transportation options vary by locality, transportation interventions need to be tailored to local needs and resources. In our study, 42% of clinics had transportation support, primarily through partner organizations, with the service more common for rural versus urban clinics. It is encouraging that clinics are attempting to address this fundamental barrier.

Clinics apply different approaches to reduce no-shows. A welcoming environment, appointment reminders (text messages and/or phone calls), and streamlined admissions were the top reported practices to reduce missed appointments. This is similar to the common strategies identified by other research to reduce no-show rates [5, 11, 12, 25, 26]. Such strategies are relatively low cost to implement and maintain compared to provision of support services to address underlying patient barriers that may be impacting ability to access and utilize healthcare services. However, nudge messages in an appointment reminder letter do not appear to have any additional benefit in reducing no-show appointments for primary or mental health care appointments in the Veterans Affairs setting [27].

The COVID-19 pandemic fundamentally impacted primary care clinic functioning. One long-term impact of the pandemic is likely to be the increased use of telehealth services, which may help address some of the time and transportation issues associated with receipt of care, assuming that patients have access to the internet at sufficient speeds, a device on which to conduct the telehealth appointment, and the technological know-how to use the service [28, 29]. Telehealth was the most common support service provided by clinics in our survey.

The major strength of this study was the sampling frame– surveys were sent to all primary care clinics throughout the state, and we were able to link survey information with the database maintained by HPTS. We had a 20% response rate, which limits the generalizability of the findings. Additionally, data was collected within a single state. Due to differences in state policies that my affect healthcare regulation and access (e.g., scope of practice, Medicaid eligibility), results may differ in other states. We did not have information about the model of care delivery or institutional funding structure of the clinics (i.e., federally qualified health center, private clinic, part of a hospital system or large healthcare network), which was another limitation of our data. Data collection started as COVID-19 cases initially surged in the US, and was then put on hold for nearly a year. The survey instrument was revised during the interim period, and data quality due to missingness was a challenge, so the level of detail about clinical populations was excluded from the planned analyses. There was also potential misclassification when we recategorized round 1 responses to accommodate the question rewording in round 2 (for example rank ordering of perceived barriers was transformed into a dichotomous “yes/no” response). Including a primary care clinic office manager on the study team and pilot testing the survey might have eliminated some of the data collection issues that we experienced.

Despite these limitations, the results of the study highlight the need to implement targeted programs that mitigate patients’ underlying needs which may lead to missed healthcare appointments. Implementing strategies that address patient barriers to attendance is a key to reducing patient no-show rates [5]. While clinics may not have the personnel and resources to offer patient support services directly, several rural clinics appear to be partnering with outside organizations to help link patients with needed services. Such efforts could become a model for other clinics to develop a more wholistic, community-centered approach to improve health care access.

## Conclusions

Primary care clinics’ perceptions of barriers their patients face in accessing timely healthcare include finances, time off work, and remembering to schedule. However, these perceived barriers do not align well with the provision of supportive services to address these needs. Providers utilize several approaches to reduce missed appointments, including a variety of reminder systems. Rural clinics appeared to have more community partnerships to address underlying social determinants of health, such as transportation and assistance applying for government aid. Taking such a wholistic partnership approach is an area for future study to improve patient access.

### Electronic supplementary material

Below is the link to the electronic supplementary material.

Supplementary Material 1

Supplementary Material 2

Supplementary Material 3

## Data Availability

The dataset used and analyzed during the current study is available from the corresponding author on reasonable request.

## Abbreviations

CI: confidence intervals
HPTS: Health Professions Tracking Service
OR: odds ratio
REDCap: Research Electronic Data Capture
RUCA: Rural-Urban Commuting Area
SD: standard deviation
