# Supplementary material for: Cross-sectional analysis of primary care clinics’ policies, practices, and availability of patient support services during the COVID-19 pandemic
Source: BMC Health Serv Res. 2024 Mar 5;24:279. doi: 10.1186/s12913-024-10660-6 (PMC10916250; doi:10.1186/s12913-024-10660-6)
Supplement: Supplementary file 1 — Supplementary Material 1 [file 12913_2024_10660_MOESM1_ESM.pdf]

Patient Access to Health Care

1. What factors seem to most impact your patients' ability to receive timely access to health care? *Please rank the following items with 1 being the most impactful, and 9 being the least impactful.*
   - _____ Clinic hours (not open in evening, weekends, etc.)
   - _____ Finances (insurance coverage, copayments, etc.)
   - _____ Language barriers
   - _____ Patients can't afford to take time off from work
   - _____ Patients can't get an appointment soon enough (not enough providers)
   - _____ Patients forget to schedule (annual visit, follow-ups, etc.)
   - _____ Patients lack dependent care (childcare, eldercare, etc.)
   - _____ Transportation
   - _____ Wait times in office to see provider
   - _____ Other (please specify): ____

Patient Resources

2. Please indicate whether any of the following services are available on-site or through a community partner:

		On-site	Partner	Not Available
a.	Patient navigator or care coordinator	☐	☐	☐
b.	Telehealth options	☐	☐	☐
	If *on-site* or *partner*, please describe: ____ ____			
c.	Social support			
	i. Social Work	☐	☐	☐
	ii. Legal Aid	☐	☐	☐
	iii. Assistance applying for Medicaid, Medicare, Supplemental Nutritional Assistance Program, etc.	☐	☐	☐
	iv. Transportation services	☐	☐	☐
	v. Dependent care services (child, elder, etc.)	☐	☐	☐

Policies & Procedures

3. Does your practice have a policy for late arrivals? ☐ Yes ☐ No

   a. If *yes*, please explain: ____

   ____

   ____

4. Does your practice have a policy for missed appointments? ☐ Yes ☐ No

   a. If *yes*, please explain: ____

   ____

   ____

5. For your clinic, how much are missed appointments a problem?

   ☐ Not at all ☐ Slightly ☐ Somewhat ☐ Very much

6. Please indicate which, if any, of the following procedures or practices are used to reduce missed appointments:

a. Overbooking to reduce office wait times	☐ Yes ☐ No
b. Set-aside walk-in appointment slots	☐ Yes ☐ No
c. Increased provider capacity within the past 2 years	☐ Yes ☐ No
d. Behavioral engagement strategy	
Motivational interviewing	☐ Yes ☐ No
Contingency management	☐ Yes ☐ No
e. Scheduling reminders	
Telephone (voice message)	☐ Yes ☐ No
Text	☐ Yes ☐ No
Email	☐ Yes ☐ No
Mail (postcard, letter)	☐ Yes ☐ No
f. Appointment reminders	
Telephone (voice message)	☐ Yes ☐ No
Text	☐ Yes ☐ No
Email	☐ Yes ☐ No
Mail (postcard, letter)	☐ Yes ☐ No
g. Streamlined admissions/check-in	☐ Yes ☐ No
h. Welcoming environment	☐ Yes ☐ No
i. Extended hours	
Evening hours	☐ Yes ☐ No
Weekend	☐ Yes ☐ No
j. Fee charged to patients for	
Late arrival	☐ Yes ☐ No
Late cancellation (i.e., less than 24 hours)	☐ Yes ☐ No
No-show/missed appointment	☐ Yes ☐ No
k. Dropped from service after missed appointments	☐ Yes ☐ No
If *yes,* specify number of missed appointments _____	

2019 Clinic Characteristics

For the following questions, please provide data for ***January 1 – December 31, 2019*** *unless otherwise specified. Please indicate whether data provided are exact numbers (based on electronic medical records, billing, or another method of regular tracking) or estimates.*

		Yes	No
7.	Does your clinic utilize Electronic Medical Records (EMR)?	☐	☐
	a. If *yes*, is the EMR used to verify patient status (up-to-date, due, past due) for:		
	i. vaccines?	☐	☐
	ii. cancer screening?	☐	☐

		#	Exact	Estimate
8.	How many patients were seen by your practice in 2019?	______	☐	☐
9.	How many of those patients were seen for an annual physical (well-being check)?	______	☐	☐
10.	How many patients were seen more than once (i.e., had a follow-up appointment or returned for a new issue) during 2019?	______	☐	☐
11.	How many appointments were scheduled			
	a. in **February 2019?**	______	☐	☐
	b. in **April 2019?**	______	☐	☐
12.	How many scheduled appointments were missed ("no show")			
	a. in **February 2019?**	______	☐	☐
	b. in **April 2019?**	______	☐	☐
13.	How many scheduled appointments were canceled less than 24 hours in advance			
	a. in **February 2019?**	______	☐	☐
	b. in **April 2019?**	______	☐	☐

		%	Exact	Estimate
14.	**Percent** of patients by insurance status:			
	a. Medicaid	______	☐	☐
	b. Medicare (any type)	______	☐	☐
	c. Private insurance	______	☐	☐
	d. Uninsured or self-pay	______	☐	☐

		Yes	No
15.	Does your facility routinely track (e.g., as Quality Control or Quality Improvement) patient compliance with recommended vaccinations?	☐	☐

15. a. If *yes*, what **percent** of your patients are up-to-date for the following:

	**%**	**Exact**	**Estimate**
i. Measles, Mumps & Rubella (MMR)	_____	☐	☐
ii. Polio (IPV)	_____	☐	☐
iii. Diphtheria, Tetanus & Pertussis (DTaP)	_____	☐	☐
iv. Varicella	_____	☐	☐
v. Haemophilus influenzae type B (Hib)	_____	☐	☐
vi. Hepatitis A	_____	☐	☐
vii. Hepatitis B	_____	☐	☐
viii. Influenza (annual)	_____	☐	☐
ix. Human papillomavirus (HPV)	_____	☐	☐

16. Does your facility routinely track (e.g., as Quality Control or Quality Improvement) patient compliance with recommended cancer screening?

**Yes**	**No**
☐	☐

a. If *yes*, what **percent** of your patients are up-to-date for the following screenings:

	**%**	**Exact**	**Estimate**
i. **Colon** (% adults 50-75 years old who had FOBT or FIT in past year, sigmoidoscopy in past 5 years, or colonoscopy in past 10 year):			
1. Any colorectal cancer screening	_____	☐	☐
2. FOBT or FIT in past year	_____	☐	☐
3. Sigmoidoscopy in past 5 years	_____	☐	☐
4. Colonoscopy in past 10 years	_____	☐	☐
ii. **Breast** (% females 50-74 with mammogram in past 2 years)	_____	☐	☐
iii. **Cervical** (% females 21-65 without hysterectomy who had Pap smear in past 3 years, or women age 30-65 who had Pap smear and HPV test within the past 5 years)	_____	☐	☐

17. Would your organization like to be included in collaborative intervention research to improve patient health outcomes?

**Yes**	**No**
☐	☐

Please provide information about the person(s) completing this form.

18. Job title: ____

19. Length of time at this organization: _______________ (years)
