# Supplementary material for: Cross-sectional analysis of primary care clinics’ policies, practices, and availability of patient support services during the COVID-19 pandemic
Source: BMC Health Serv Res. 2024 Mar 5;24:279. doi: 10.1186/s12913-024-10660-6 (PMC10916250; doi:10.1186/s12913-024-10660-6)
Supplement: Supplementary file 2 — Supplementary Material 2 [file 12913_2024_10660_MOESM2_ESM.pdf]

Additional File 2: Round 2 mailed survey
Patient Barriers to Accessing Health Care: Nebraska Primary Care Providers' Perspectives

Patient Access to Health Care

1. What factors seem to most impact your patients' ability to receive timely access to health care? ***Please mark all that apply***.
   - _____ Clinic hours (not open in evening, weekends, etc.)
   - _____ Finances (insurance coverage, copayments, etc.)
   - _____ Language barriers
   - _____ Patients can't afford to take time off from work
   - _____ Patients can't get an appointment soon enough (not enough providers)
   - _____ Patients forget to schedule (annual visit, follow-ups, etc.)
   - _____ Patients lack dependent care (childcare, eldercare, etc.)
   - _____ Transportation
   - _____ Wait times in office to see provider
   - _____ Other (please specify): ____

Patient Resources

2. Please indicate whether any of the following services are available on-site or through a community partner:

	**On-site**	**Partner**	**Not Available**
a. Patient navigator or care coordinator	☐	☐	☐
b. Telehealth options	☐	☐	☐

If *telehealth* is available, please describe: ________________________________

____

____

	**On-site**	**Partner**	**Not Available**
c. Social support			
i. Social Work	☐	☐	☐
ii. Legal Aid	☐	☐	☐
iii. Assistance applying for Medicaid, Medicare, Supplemental Nutritional Assistance Program, etc.	☐	☐	☐
iv. Transportation services	☐	☐	☐
v. Dependent care services (child, elder, etc.)	☐	☐	☐

Policies & Procedures

3. Does your practice have a policy for late arrivals? ☐ Yes ☐ No

   a. If *yes*, please explain: ____

   ____

   ____

4. Does your practice have a policy for missed appointments? ☐ Yes ☐ No

   a. If *yes*, please explain: ____

   ____

   ____

5. For your clinic, how much are missed appointments a problem?

   ☐ Not at all ☐ Slightly ☐ Somewhat ☐ Very much

6. Please indicate which, if any, of the following procedures or practices are used to reduce missed appointments:

   a. Overbooking to reduce office wait times ☐ Yes ☐ No
   b. Set-aside walk-in appointment slots ☐ Yes ☐ No
   c. Increased provider capacity within the past 2 years ☐ Yes ☐ No
   d. Behavioral engagement strategy
      Motivational interviewing ☐ Yes ☐ No
      Contingency management ☐ Yes ☐ No
   e. Scheduling reminders
      Telephone (voice message) ☐ Yes ☐ No
      Text ☐ Yes ☐ No
      Email ☐ Yes ☐ No
      Mail (postcard, letter) ☐ Yes ☐ No
   f. Appointment reminders
      Telephone (voice message) ☐ Yes ☐ No
      Text ☐ Yes ☐ No
      Email ☐ Yes ☐ No
      Mail (postcard, letter) ☐ Yes ☐ No
   g. Streamlined admissions/check-in ☐ Yes ☐ No
   h. Welcoming environment ☐ Yes ☐ No
   i. Extended hours
      Evening hours ☐ Yes ☐ No
      Weekend ☐ Yes ☐ No
   j. Fee charged to patients for
      Late arrival ☐ Yes ☐ No
      Late cancellation (i.e., less than 24 hours) ☐ Yes ☐ No
      No-show/missed appointment ☐ Yes ☐ No
   k. Dropped from service after missed appointments ☐ Yes ☐ No
      If *yes,* specify number of missed appointments _____

2019 & 2020 Clinic Characteristics

*We want to compare clinic usage before and during the COVID-19 pandemic. For the following questions, please provide data for **2019** and for **2020 (January 1-September 30, 2020)**. Please indicate whether data provided are an estimate rather than exact numbers (based on electronic medical records, billing, or another method of regular tracking).*

	Yes	No
7. Does your clinic utilize Electronic Medical Records (EMR)?	☐	☐
a. If *yes*, is the EMR used to verify patient status (up-to-date, due, past due) for:		
i. vaccines?	☐	☐
ii. cancer screening?	☐	☐

	2019	2020	Mark if this is an estimate
8. How many patients were seen by your practice?	_____	_____	☐
9. **Percent** of patients by insurance status:			
a. Medicaid	_____	_____	☐
b. Medicare (any type)	_____	_____	☐
c. Private insurance	_____	_____	☐
d. Uninsured or self-pay	_____	_____	☐

	Yes	No
10. Does your facility routinely track (e.g., as Quality Control or Quality Improvement) patient compliance with recommended cancer screening?	☐	☐

	2019	2020	Mark if this is an estimate
a. If *yes*, what **percent** of your patients were up-to-date for the following screenings:			
i. **Colon** (% adults 50-75 years old who had FOBT or FIT in past year, sigmoidoscopy in past 5 years, or colonoscopy in past 10 year):	_____	_____	☐
ii. **Breast** (% females 50-74 with mammogram in past 2 years)	_____	_____	☐
iii. **Cervical** (% females 21-65 without hysterectomy who had Pap smear in past 3 years, or women age 30-65 who had Pap smear and HPV test within the past 5 years)	_____	_____	☐

	Yes	No
11. Would your organization like to be included in collaborative intervention research to improve patient health outcomes?	☐	☐

COVID-19 Clinic Impacts on Health Care Access

Compared to 2019, during March-May 2020…	Decreased	Unchanged	Increased
12. the number of patients seen …	☐	☐	☐
13. our operating hours …	☐	☐	☐
14. our staff availability …	☐	☐	☐
15. our telehealth services …	☐	☐	☐
**Compared to 2019, since June 2020…**			
16. the number of patients seen …	☐	☐	☐
17. our operating hours …	☐	☐	☐
18. our staff availability …	☐	☐	☐
19. our telehealth services …	☐	☐	☐

20. To what extent has COVID-19 impacted your organization's ability to provide health services?

☐ No impact ☐ Slight impact ☐ Moderate impact ☐ Major impact

21. **If moderate or major impact**, please describe: ____________________________________

____

____

22. How satisfied are you with your patients' ability to receive timely COVID testing?

☐ Very satisfied ☐ Somewhat satisfied ☐ Neither satisfied nor dissatisfied ☐ Somewhat dissatisfied ☐ Very dissatisfied

23. Please share with us anything else you would like us to know about the impact of COVID-19 on your patients or practice. ____

____

____

Please provide information about the person(s) completing this form.

24. Job title: ____

25. Length of time at this organization: _______________ (years)
