# Supplementary material for: Cross-sectional analysis of primary care clinics’ policies, practices, and availability of patient support services during the COVID-19 pandemic
Source: BMC Health Serv Res. 2024 Mar 5;24:279. doi: 10.1186/s12913-024-10660-6 (PMC10916250; doi:10.1186/s12913-024-10660-6)
Supplement: Supplementary file 3 — Supplementary Material 3 [file 12913_2024_10660_MOESM3_ESM.docx]

**Additional File 3. Analysis of Non-Response Bias**

| **Clinic Characteristic**  n (%) or mean (SD) | **Overall**  **n=577** | **Response**  **n=99** | **No Response**  **n=478** | **OR or Mean Difference†**  **(95% CI)** | **P-value** |
| --- | --- | --- | --- | --- | --- |
| Rurality |  |  |  | 2.31 (1.49 - 3.59) | <0.001 |
| Rural | 221 (38.3) | 55 (55.5) | 166 (34.7) |  |  |
| Urban | 351 (60.8) | 44 (44.4) | 307 (64.2) |  |  |
| Missing | 5 (0.9) | 0 | 5 (1.0) |  |  |
| Has satellite location |  |  |  | 0.83 (0.52 - 1.33) | 0.44 |
| Yes | 188 (32.6) | 29 (29.3) | 159 (33.3) |  |  |
| No | 389 (67.4) | 70 (70.7) | 319 (66.7) |  |  |
| Family medicine specialty |  |  |  | 1.24 (0.78 - 1.97) | 0.35 |
| Yes | 367 (63.6) | 67 (67.7) | 300 (62.8) |  |  |
| No | 210 (36.4) | 32 (32.3) | 178 (37.2) |  |  |
| Number of providers at primary location | 4.3 (4.6) | 4.2 (4.1) | 4.3 (4.7) | -0.12 (-1.12 - 0.89) | 0.82 |

Note: Missing data was excluded from calculating the odds ratio

†Odds ratio (OR) was calculated for categorical variables, mean difference was calculated for continuous variable.
